# Algorithms for Weld Depth Measurement in Laser Welding of Copper with Scanning Optical Coherence Tomography

**DOI:** 10.3390/mi13122243

**Published:** 2022-12-16

**Authors:** Thomas Will, Eduardo Massieu Garcia, Claudio Hoelbling, Christian Goth, Michael Schmidt

**Affiliations:** 1 Institute of Photonic Technologies, Friedrich-Alexander Universität Erlangen-Nürnberg, 91052 Erlangen, Germany; 2 Erlangen Graduate School in Advanced Optical Technologies (SAOT), Friedrich-Alexander Universität Erlangen-Nürnberg, 91052 Erlangen, Germany; 3 Vitesco Technologies Germany GmbH, 90411 Nürnberg, Germany

**Keywords:** optical coherence tomography, interferometry, laser welding, copper, process monitoring, quality monitoring, data processing

## Abstract

In-process monitoring of weld penetration depth is possible with optical coherence tomography (OCT). The weld depth can be identified with OCT by statistical signal processing of the raw OCT signal and keyhole mapping. This approach is only applicable to stable welding processes and requires a time-consuming keyhole mapping to identify the optimal placement of a singular OCT measuring beam. In this work, we use an OCT measurement line for the identification of the weld depth. This approach shows the advantage that the calibration effort can be reduced as the measurement line requires only calibration in one dimension. As current literature focuses on weld depth measurement with a singular measurement point in the keyhole, no optimal algorithm exists for weld depth measurement with an OCT measurement line. We developed seven different weld depth processing pipelines and tested these algorithms under different weld conditions, such as stable deep penetration welding, unstable deep penetration welding, and heat conduction welding. We analyzed the accuracy of the weld depth processing algorithms by comparing the measured weld depth with metallographic weld depths. The intensity accumulation approach is identified as the most accurate algorithm for successful weld depth measurement with a scanning OCT measurement line.

## 1. Introduction

The laser welding of copper materials gains relevance in an increasing number of applications in electronic products such as battery tab connectors [1] and stator hairpins [2]. One crucial quality indicator for the laser welding of copper is the weld penetration depth [3,4]. Exceeding the full penetration of joint partners leads to damage to the weld joint surroundings in the product. Non-sufficient weld penetration depth reduces the connection area and hence can be the reason for a malfunction. One solution to observe the weld penetration depth inline is optical coherence tomography (OCT). OCT is an interferometric measurement technology that enables the measurement of weld penetration depth coaxially to the processing laser in a fixed optic [5,6,7,8,9,10,11,12] or scanning optic [13,14,15].

The successful determination of the weld penetration depth with OCT depends on the accurate positioning of the measurement beam in the keyhole, and the choice of a suitable OCT data processing approach. The keyhole mapping approach was suggested to determine optimal measurement beam placement [7]. This approach applies an iterative two-dimensional adjustment of measurement position until the optimal position is found. Accurate measurement of the weld penetration depth requires the position of the measurement beam as close to the bottom of the keyhole as possible [1] to achieve a direct back reflection. A tilted keyhole wall may lead to an inappropriate reflection angle and hence a reduced signal intensity [16] that reduces the discriminability of useful signal from noise. In consequence, experiments are performed with a changing measurement beam position in the keyhole until an optimal placement is found [1]. Such alignment is not universal and needs to be adjusted for each welding task with respect to the keyhole shape [3,17]. For instance, the higher the welding speed and the higher the laser power, the higher the distance between OCT measurement beam from the tool center point (TCP) of the processing laser, in the case of the laser welding of copper [13].

The goal of the data processing approach is to identify the maximum intensity within the resulting measurement signal at each measurement point, as the signal intensity of the OCT is maximized for a direct reflection from the keyhole bottom. However, different artifacts and noise may occur that make it difficult to identify the keyhole depth position. The noise in the OCT signal originates either from the setup or the process. The setup noise includes artifacts from the DC-term [16] and the interaction of the OCT beam with lenses in the optical setup. The process noise can be caused by changes in the optical path by the vapor plume in the welding process and thermal noise [16]. To date, this issue was treated with data analysis methods such as histogram analysis [9,17], different filtering (e.g., percentile filter [9,10], Kalman filtering [14]), or kernel density estimation (KDE) [1,7]. The simplest method is the evaluation of histograms at every measurement point [17] and the extraction of the local maximum [9]. This approach can be improved by the usage of histograms for segmentation of the signal for the separation of noise from useful signal in the first instance, and in combination with a percentile filter for feature extraction [10]. The application of a running percentile filter after removing the noise can be seen as beneficial [10], where a percentile filter is applied to the measurement information along the measurement time for weld depth identification. A further increase in accuracy can be achieved with a combined usage of bandpass and Kalman filtering [14]. However, filtering requires parametrization of the relevant percentile and moving window according to the weld settings, and histograms are not deterministic as maxima could be found at measurement positions with multiple reflections.

Alternative data processing approaches are necessary to enable a more deterministic approach, while acquiring insights into the keyhole dynamics. A kernel density estimation (KDE) with Gaussian kernel applied to a moving window, with regards to the signal time-series, allows to calculate a nonparametric estimate of the statistical density function. First, the weld penetration depth can be determined with an 80^th^ percentile filter from the statistical density function [7]. Second, the distribution of the statistical density function can give feedback on the keyhole dynamics by computing the modality index with Hartigans’ dip test [7]. The statistical density function distribution can be single-modal, bi-modal, or multi-modal. A single-modal distribution can be expected for a measurement at the keyhole bottom. A bi-modal distribution results from a measurement beam that is shifted toward the keyhole wall. A multi-modal distribution may capture fluctuations in the keyhole opening with eventually multiple humps on the keyhole wall. In consequence, the measured weld penetration depth can be determined in the combination of the assessment of the modality index and the application of an 80^th^ percentile filter to the statistical density function.

Finally, the direct measurement of the weld penetration depth can be acquired by finally subtracting the result of the distance measurement from one measurement beam in the keyhole, and another on the surface plane of the workpiece [18].

Nevertheless, all the before mentioned approaches to determine the weld penetration depth using only one measurement point in the keyhole, require time-consuming keyhole mapping in two dimensions. Our work proposes the measurement of the weld penetration depth with a measurement beam that is scanned laterally to the processing laser. In consequence, we expect to reduce the effort in keyhole mapping. Furthermore, no optimal data processing approach exists for two-dimensional measurement information from laterally scanned OCT measurement beams in the case of weld penetration depth measurement.

In consequence, we investigate the applicability of seven different data processing approaches for weld depth determination for laser welding of copper with a laterally scanned OCT measurement beam at four different distances towards the TCP. The applicability of each data processing approach is determined for different weld conditions with stable and unstable keyhole, as well as welding parameters in the heat conduction regime with measurements in the melt pool denudation. The accuracy of each data processing approach is determined by a comparison of the measurement error towards metallographic weld depth measurements.

## 2. Materials and Methods

First, the experimental setup is introduced. Second, the experimental procedure is described. Lastly, the data processing approaches are described for the weld depth measurement from laterally scanned OCT measurements, and methods for the comparison of the weld depth measurement with metallographic evaluations.

### 2.1. Experimental Setup

**Figure 1:**
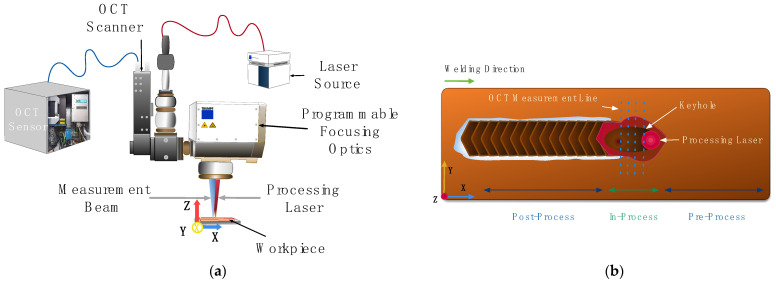
(**a**) Schematic experimental setup with OCT, processing laser, programmable focusing optics, and workpiece; (**b**) schematic of OCT measurement positions behind TCP.

The experimental setup consists of a programmable focusing optic with cross-jet, fiber-coupled processing laser, and OCT (Figure 1). The laser welding process is performed using a continuous wave disk laser (Trumpf TruDisk 6001) at a wavelength of 1030 nm with a maximum average power of 6000 W. The laser light is coupled into a programmable focusing optic (Trumpf PFO 33-2) with the help of a fiber (core diameter 100 µm). The focusing optic has a focal length of 255 mm and results in a laser spot diameter of 170 µm. The programmable focusing optic consists of galvanometer scanners and allow for scanning in an elliptical field of 90 mm × 50 mm. The OCT is attached to the programmable focusing optics and enables a coaxial positioning of the measurement beam. The OCT is an SD-OCT with a superluminescent diode with a central wavelength at 840 nm and a bandwidth of ±20 nm. The measurement beam is detected on a 2048-pixel line sensor with a maximum measurement frequency of 70 kHz. The OCT system has an axial resolution of 12 µm (z-direction). The lateral resolution is 25 µm (y-direction) and is related to the spot size of our measurement beam.

### 2.2. Experimental Procedure

A weld seam length of 60 mm is welded bead-on-plate on pure copper workpieces (Cu-OFE, 70 mm × 30 mm × 5 mm) in the focus position. During the welding process, the OCT measurement beam is scanned perpendicular to the weld seam with the welding speed in the x-direction. The OCT measurement beam has a length of 2 mm in the y-direction with 200 measurement points and resulted in a frame rate of 87 Hz (Figure 1). In consequence, we acquire one measurement point per 10 µm. This leads to a minimum number of 17 measurement points in the keyhole if we consider a keyhole width according to the focus diameter of our processing laser with 170 µm. However, the width can be bigger for unstable deep penetration welding resulting in an increased amount of sample points in the keyhole. This amount of sample points can be already considered beneficial in contrast to single-point measurements that require iterative testing to identify the keyhole bottom.

Parameters are varied for laser process parameters, and OCT parameters (Table 1). The laser process parameters are varied by process regime in the case of heat conduction welding, stable deep penetration welding, and unstable deep penetration welding with the formation of melt ejections. The process regime under investigation depends on the combination of laser power and welding speed and varies between 3000 W and 6000 W as well as 6 m/min and 60 m/min. The OCT parameters are varied in terms of the measurement position in the x-direction relative to the tool center point. The relative OCT position is chosen to be trailing due to the expected tilt of the keyhole wall. The measurement position is varied from 0.06 mm up to 0.12 mm in 0.02 mm steps as keyhole mapping in literature resulted in optimal positioning for this step size [1]. All parameters in Table 1 are repeated three times for each set of parameters.

### 2.3. Data Processing

**Figure 2:**
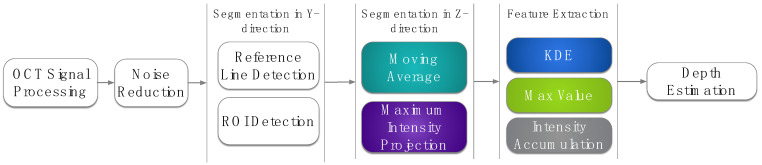
Data processing steps for the identification of weld depth with OCT measurements. Colored data processing blocks are varied.

Weld depth measurements are performed by OCT data and the metallographic evaluation of probes. Scanned OCT measurements follow the same data processing procedure: OCT sensor signal transformation to depth information, noise reduction, segmentation in y-direction, segmentation in z-direction, and feature extraction for the measurement of weld penetration depth (Figure 2). The OCT sensor signal transformation to depth information is carried out by the k-linearization of the sensing data and Fourier transformation of the measurement information.

Noise reduction is performed by background subtraction with averaged background information. Remaining noise artifacts from lenses in the optical setup are identified by Hough transform and set to zero.

Afterwards, a segmentation in the y-direction is required to distinguish between the workpiece surface and the keyhole opening. First, the workpiece surface is detected by identification of the image row with maximum accumulated signal intensity as the signal intensity on the workpiece surface is higher than the signal intensity in the keyhole. The workpiece surface determines the reference position for weld depth measurement in the keyhole. Second, the keyhole is determined by identification of columns in the y-direction with reduced signal intensity by analysis of the falling and rising edge intensity at workpiece surface level.

**Figure 3:**
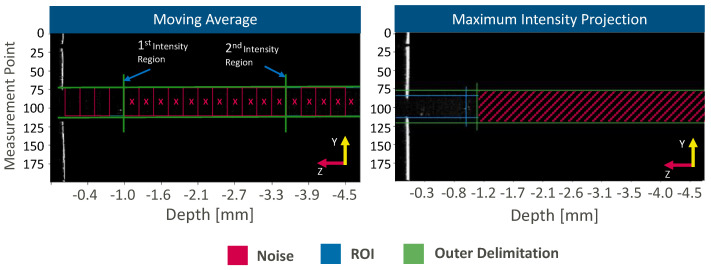
Segmentation in the z-direction. Schematic of the moving average (MA) and maximum intensity projection (MIP) approach.

Only the keyhole opening is considered for further processing steps. The segmentation of the keyhole opening in the z-direction aims for the isolation of measurement information from the keyhole bottom by separating the workpiece surface, the keyhole region, and the region below the keyhole. Two different methods are applied for this segmentation process: the moving average method [19] and the maximum intensity projection (MIP) method [20] (Figure 3).

The moving average method calculates the mean signal intensity in a window with a depth of 40 pixels in the z-direction and a width that corresponds to the width of the keyhole opening in the y-direction. Starting at the workpiece surface, the window is moved in the z-direction after each calculation up to a distance of 5 mm. This limit is set according to the thickness of the workpiece. Reflections from the keyhole bottom lead to an increase in signal intensity. In consequence, we expect windows not to contain measurement information from the keyhole bottom if they fall below the 75th percentile of all calculated mean values. A window is chosen as the lower limit of the keyhole if the percentile condition is fulfilled, and no neighboring window above the 75th percentile can be found in the negative z-direction. All windows below this window are discarded.

In contrast to the moving average method that is applied to each 2D OCT image frame, the MIP method considers the measurement information from all 2D frames along the measurement time. Here, each coordinate of a 2D OCT image frame in z- and y-direction are compared with the identical coordinate in all other taken 2D OCT image frames regarding the intensity value. Maximum intensity values of a coordinate are considered for the resulting projected image frame. The highest pixel intensity region within the projected image frame defines the keyhole region, while measurement information below this limit is discarded. The maximum width of the keyhole opening defines the outer limit in the y-direction.

**Figure 4:**
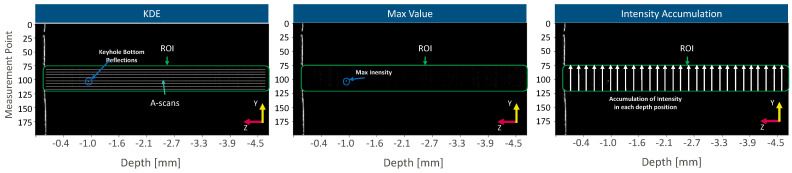
Feature extraction for weld depth identification. Schematic of the applied three different feature extraction methods (KDE, max value, intensity accumulation (IA)).

The measurement of weld penetration depth is tested with three different feature extraction algorithms: intensity accumulation, max-value, and kernel density estimation. These approaches for feature extraction differently consider the dimensionality of the measurement information (Figure 4).

The application of KDE is an adaption from the literature [1]. Here, each measurement point in the y-direction within the keyhole region is considered as a singular keyhole mapping measurement point and calculates the KDE and the modality index with the help of Hartigan’s dip test. According to the literature, only single-modal measurement points are considered for further weld penetration depth extraction. Finally, measurement points that fulfill the single-modal condition are analyzed by an 80th percentile filter along the z-direction resulting in the pixel value at the expected weld penetration depth.

The accumulation of intensity differs from the application of KDE as the lateral measurement information is seen as a whole in the y-direction. The accumulation of intensity approach follows two steps: thresholding and the accumulation of intensity. Thresholding is performed by the identification of each maximum intensity value within each measurement point in the y-direction and subsequent application of an 80th percentile. This percentile filter is chosen as measurement points that are directed towards keyhole walls show lower intensity values than a measurement point directed in the keyhole bottom. Afterwards, the weld penetration depth is extracted by an accumulation of intensity. Here, the intensity values for all lateral measurement points in the y-direction are added at each z-coordinate in the keyhole. This process is repeated for the subsequent 2D-OCT image frames. The z-position with the maximum accumulated intensity is considered the weld penetration depth. Finally, the max-value approach is the simplest feature extraction method. Here, the weld penetration depth is determined by extraction of the maximum signal intensity value within the keyhole. In the end, the extracted pixel coordinate in the z-direction is transformed to the weld penetration depth by considering the axial pixel size of 11.7 µm.

In this study, a comparison of the different segmentation and feature extraction algorithms is performed. The analysis of the different data processing methods is possible by comparing measured weld penetration depths with weld penetrations depths that are determined via metallographic cross-sections. The comparison between measured weld penetration depth by OCT and metallographic analysis are performed by root mean square error (RMSE).

The RMSE can be calculated as follows:

(1) $$RMSE=\sqrt{\frac{\sum_{i=1}^{n}{\left(y_{i}-\hat{y}\right)}^{2}}{n}}$$

where *n* is equal to the total number of in-process frames, *y_i_* is the algorithm’s calculated weld seam depth, and $\hat{y}$ the mean weld seam depth of the metallographic analysis. The higher the RMSE value, the higher the deviations from the metallographic weld seam depth of an OCT measurement. The maximum deviation allowed is the standard deviation of the metallographic weld seam depth of the corresponding welding parameters.

## 3. Results and Discussion

The goal of our work is to identify the best data processing approach for laterally scanned OCT measurement beams for weld penetration depth measurements, and to show the applicability of laterally scanned OCT measurement beams for weld depth determination. In consequence, we analyze the applicability of our different data processing approaches for stable deep penetration welding, unstable deep penetration welding, and heat conduction welding.

### 3.1. Deep Penetration Welding in a Stable Process

**Figure 5:**
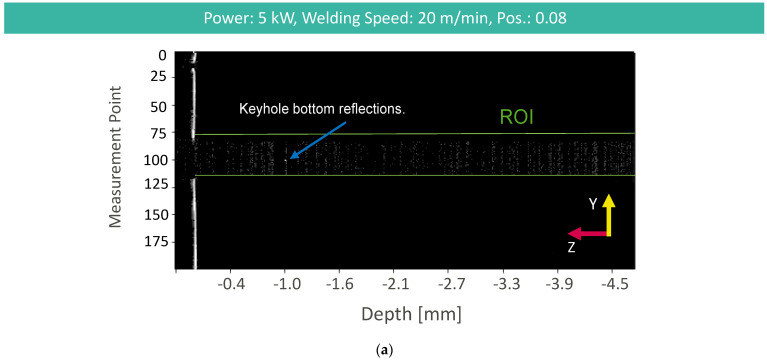

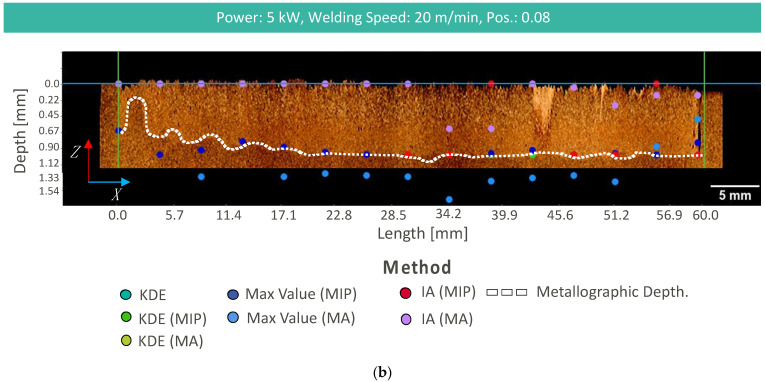
(**a**) OCT image of the weld depth measurement for stable deep penetration welding; (**b**) resulting weld depth measurement for each data processing method with longitudinal cross-section of the stable deep penetration weld.

**Figure 6:**
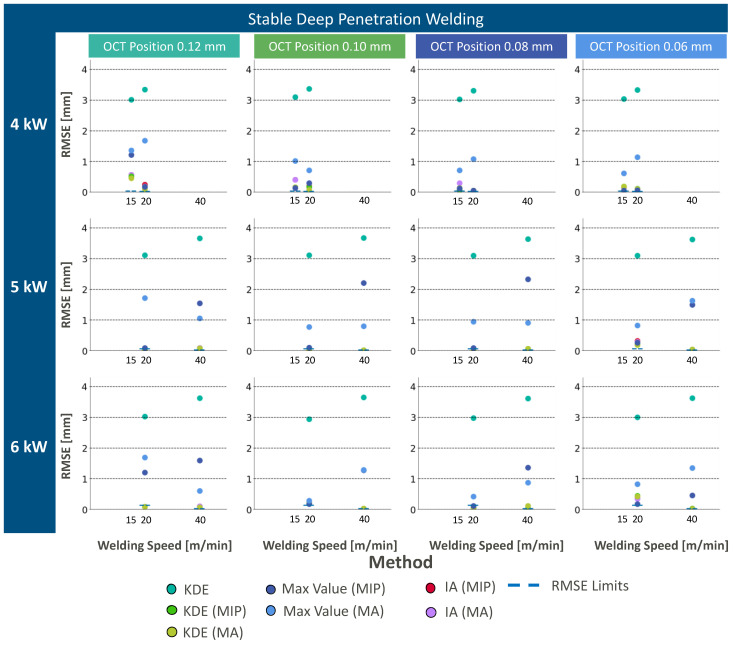
Resulting RMSE values for different applied laser and OCT parameters as well as different data processing approaches in the case of stable deep penetration welding.

OCT measurement information in the case of stable deep penetration welding processes shows characteristically increased noise in the keyhole region, due to the influence of the vapor plume and the high reflectivity of the molten material, with a maximum signal intensity within the ROI at the keyhole bottom (Figure 5a). We applied different combinations of z-direction segmentation approaches and weld depth extraction methods (Figure 5b). Data processing results can be found in Figure 5b and Figure 6 for KDE (green), max intensity (blue), and IA (red) for all four different OCT measurement positions and different segmentation methods. The RMSE limit results from the difference between the maximum and minimum metallographic weld seam depth of the corresponding welding parameters. Measurement results below this limit can be considered good weld depth measurements. Measurement results above this limit can be considered as not successful weld depth measurements.

Overall, the influence of the OCT measurement beam position behind the TCP within the range of 0.06 mm and 0.12 mm does not show a significant impact on the capability of the different data processing approaches in the case of stable deep penetration welding. The combination of the laser power and the welding speed shows a higher impact on the overall measurement success. Welding at higher laser powers (e.g., 5000 W, 6000 W) shows generally more successful weld depth measurements in comparison to welding at 4000 W. This can be explained by the change in the keyhole morphology, where possibly a different measurement position is required to identify the keyhole bottom. As all data processing methods show a trend towards lower RMSE value for an increase in the OCT measurement position behind the TCP, at a laser power of 4000 W and a welding speed of 15 m/min, we expect a more accurate weld depth measurement for these parameters for a further increase in OCT measurement position behind the TCP. This is in accordance with findings by Beck et al. [13] who could show that an alteration in laser power and welding speed leads to a change in the OCT measurement beam position behind the TCP.

As no accurate measurement of the weld depth is possible for welding at 4000 W and 15 m/min within our range of OCT measurement positions, we focus on welding at 5000 W and 6000 W at 20 m/min and 40 m/min for the comparison of the different data processing approaches. Generally, a standalone application of KDE without z-direction segmentation does not lead to accurate weld depth measurements (Figure 6). This is due to remaining static noise artifacts at measurement positions deeper than the weld depth. In consequence, the 80th percentile filter overestimates the measured weld depth and makes the application of z-direction segmentation crucial. This can be considered to be relevant for any other weld depth extraction approach, as the remaining artifacts may impact the measurement results. The application of segmentation methods such as MIP and MA improves the accuracy of the weld depth extraction methods. In the case of KDE, the best results could be achieved in the combination with MIP, whereas the application of MA leads to higher RMSE values. MIP in combination with KDE shows a better performance than MA in combination with KDE, because of the smaller ROI size in the z-direction closer to the keyhole depth. As KDE performs an 80th percentile filtering, the length in the z-direction leads to better RMSE values for segmentation with MIP, while also artifacts below the keyhole depth are cut-off. The consideration of this segmentation approach is an add-on to the described methods by Sokolov et al. [7] and shows its feasibility for laterally scanned OCT measurement beams without the need for keyhole mapping in an additional dimension.

Similar results can be achieved with max intensity extraction. As the extraction of maximum intensity requires lower computational effort than the calculation of an 80th percentile, the maximum intensity weld depth extraction can be considered as a good alternative to the established KDE weld depth extraction. However, the max intensity weld depth extraction is sensitive to measurement positions at the keyhole side walls, where also maximum intensity information can be found due to the specific keyhole morphology in the case of the laterally scanned OCT measurement beam.

Intensity accumulation as a weld depth extraction method overcomes this issue. The keyhole bottom has a certain width that leads to higher neighboring pixel intensities. As the feature extraction with intensity accumulation weighs this aspect of measurement information, improved RMSE values can be found for IA regardless of the segmentation method. In consequence, IA can be recommended as a weld depth extraction approach for laterally scanned OCT measurement beams.

### 3.2. Deep Penetration Welding in an Unstable Process

**Figure 7:**
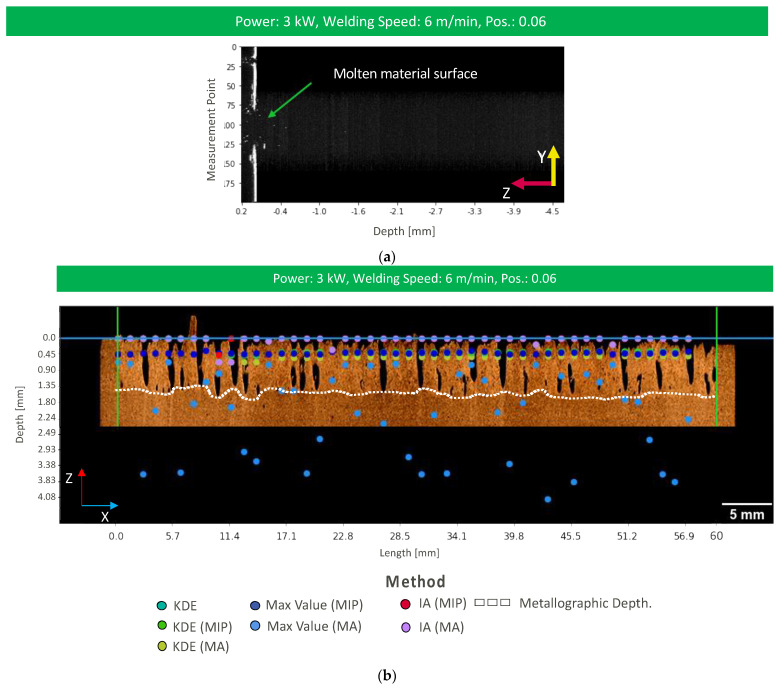
(**a**) OCT image of the weld depth measurement for unstable deep penetration welding; (**b**) resulting weld depth measurement for each data processing method with a longitudinal cross-section of the unstable deep penetration weld.

**Figure 8:**
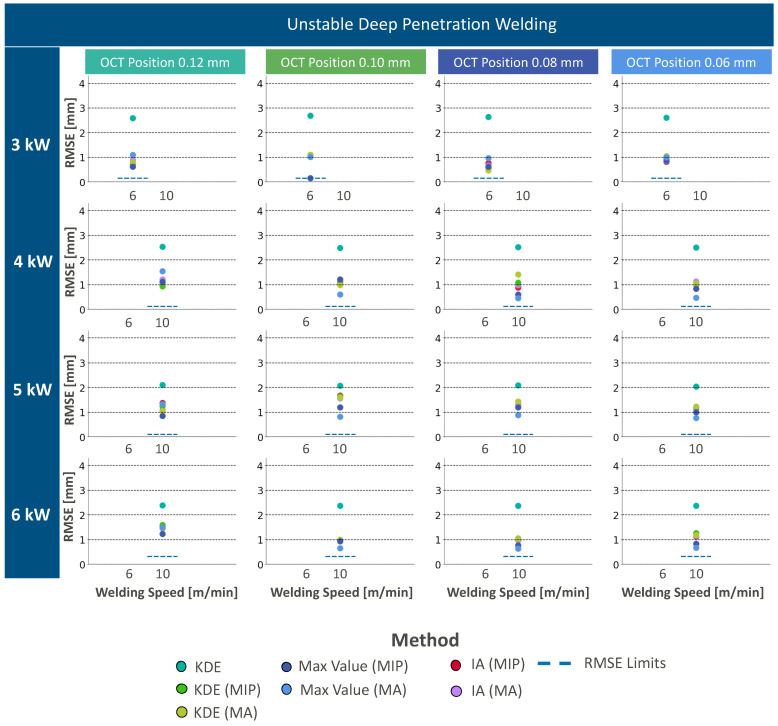
Resulting RMSE values for different applied laser and OCT parameters as well as different data processing approaches in the case of unstable deep penetration welding.

OCT measurement information in the case of unstable deep penetration welding processes shows similar noise in the keyhole region like stable deep penetration welding. Additionally, the beginning of melt ejections in the form of spatters can be recognized within OCT measurements (Figure 7a). All data processing approaches are applied to the given OCT data. Data processing results can be found in Figure 7b and Figure 8 for KDE (green), max intensity (blue), and IA (red) for all four different OCT measurement positions and different segmentation methods.

The application of KDE without z-direction segmentation is the only data processing pipeline that leads to an overestimation of the weld depth (Figure 2c) because of the remaining static noise artifacts at measurement positions deeper than the weld depth. In consequence, the 80th percentile filter without segmentation remains inapplicable also for unstable deep penetration welding.

The application of segmentation methods, such as MIP and MA, improves the accuracy of the weld depth extraction method and tends to the identification of underestimated weld depths (Figure 8). The underestimation leads to the conclusion that the measurement beam cannot be directed on the keyhole bottom, and is rather positioned on a bulge of molten material. Interestingly, the RMSE values for KDE-based weld depth extraction tend to be lower than RMSE values with weld depth extraction by intensity accumulation. This is the case as the intensity accumulation approach sums up the values in lateral direction and evokes higher RMSE values as the measurement information rather contains information from the dynamic molten material surface at different depth positions. In consequence, the weld depth is rather underestimated.

Max intensity extraction shows the lowest resulting RMSE values in comparison to the other weld depth extraction methods. Here, we expect that this weld depth extraction method results in the lowest RMSE values as this approach does not show a directional dependence towards the measurement information. Here, the maximum intensity value allows concluding on direct reflections from the keyhole morphology. Measurement errors may result from direct reflection at keyhole positions closer to the workpiece surface.

The choice of the segmentation method does not show a significant impact on the RMSE value regardless of the feature extraction method. This is the case as the relevant measurement information is extracted closer to the workpiece surface due to high signal intensity components that originate in reflections from the keyhole morphology at elevated positions instead of the keyhole bottom. In consequence, the relevance of the ROI size in the z-direction and the resulting influence by artifacts at positions below the keyhole bottom are negligible.

In conclusion, the unstable keyhole does not allow for accurate measurements of the weld depth. Weld depth extraction methods enable to identify the keyhole morphology but presumably fail to identify the keyhole bottom due to the dynamic melt flow. Best results are achieved with the max intensity extraction as this approach does not show a directionality in its detection of relevant measurement information in the keyhole.

### 3.3. Heat Conduction Welding

**Figure 9:**
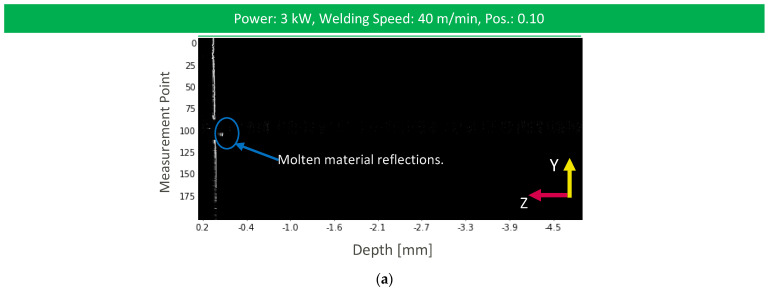

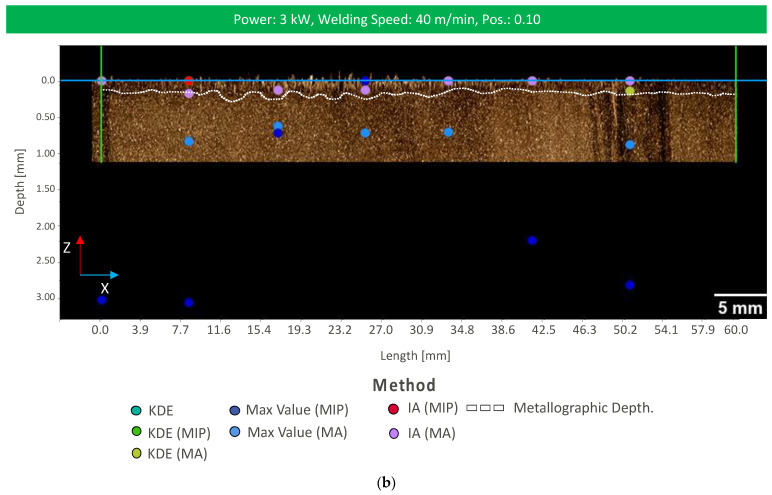
(**a**) OCT image of the weld depth measurement for unstable deep penetration welding; (**b**) resulting weld depth measurement for each data processing method with a longitudinal cross-section of the heat conduction weld.

**Figure 10:**
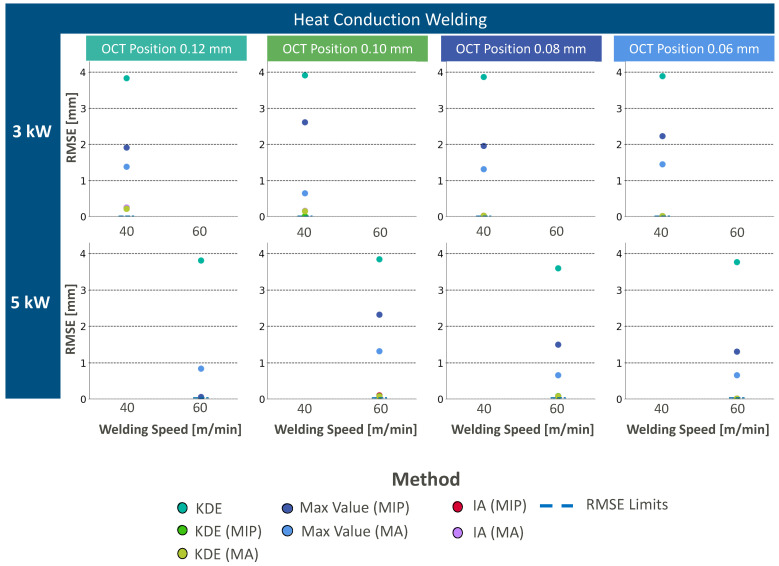
Resulting RMSE values for different applied laser and OCT parameters as well as different data processing approaches in the case of heat conduction welding.

OCT measurement information in the case of heat conduction welding processes shows slightly less noise in the interaction region as no vapor plume is generated as in stable deep penetration welding (Figure 9a). However, like the before mentioned welding process, reduced measurement information is found in the interaction region with reduced signal intensity in comparison to the workpiece surface. This is the result of the OCT measurement position on the molten material that resembles a structure with very low roughness and reduces the amount of reflected light to the sensor. Nevertheless, material reflections can be found below the workpiece surface level. Here, we expect that laser welding with scanning optics may lead to a melt flow with a denudation along the weld due to the changing processing laser beam angle. This denudation can give feedback on the weld penetration depth. Most of our data processing approaches resulted in weld depth measurements with RMSE values that can give feedback about the resulting weld penetration depth (Figure 9b and Figure 10).

Like previously mentioned results, experimental results for heat conduction welding by the weld penetration depth extraction approach with KDE show the highest RMSE values without previous segmentation in the z-direction (Figure 10). As soon as the segmentation in the z-direction is within the data processing pipeline, the weld depth can be successfully determined with both segmentation approaches. The MIP-based segmentation approach shows lower RMSE values than the MA-based segmentation approach. This results from the differences in ROI length and would require an adjustment of the percentile filter for improvements. Weld depth feature extraction with max intensity extraction leads to low RMSE values for data processing pipelines with segmentation with MIP. The segmentation with MA leads to higher RMSE values with max intensity extraction as possibly again due to the identification of remaining artifacts. The lowest RMSE values can be found for the data processing pipeline with intensity accumulation. No significant differences can be found for the application of MA or MIP for segmentation.

In conclusion, similar findings with regards to the data processing approaches can be identified for heat conduction welding, as for stable deep penetration welding. The application of intensity accumulation results in the lowest RMSE values due to the measurement information from the denudation bottom at different lateral positions.

## 4. Conclusions

In this report, experimental work was performed to assess the influence of different data processing approaches for weld depth measurements from laterally scanned OCT data in the case of laser welding copper. It was shown that the segmentation of the OCT data in the z-direction is crucial for successful weld depth extraction. The MIP segmentation approach showed better RMSE values than the MA segmentation approach with all further weld depth extraction methods. A successful weld depth extraction was possible by intensity accumulation within the ROI of the laterally scanned OCT measurement beam for heat conduction welding and deep penetration welding. Overall, the extraction of the maximum intensity shows a good alternative to KDE. The disadvantage of KDE and maximum intensity as weld depth extraction methods, in comparison to the intensity accumulation method, are based on the higher sensitivity to measurement information extraction from measurement points that are not directed to the keyhole bottom. Measurements with unstable deep penetration welds resulted in non-successful weld depth extractions due to the high melt pool dynamics. We expect these findings to be beneficial for quality assessment in laser welding. The shown applicability of different data processing approaches for weld depth extraction of a laterally scanned OCT measurement beam will reduce the calibration effort for weld depth measurements with OCT, and shows alternative data pipeline in comparison to the state-of-the-art.

## Figures and Tables

**Table 1:** OCT and laser welding parameters with parameter values.

OCT Parameters	Parameter Value	
Measurement position behind TCP ∆_x_ (mm)	0.06, 0.08, 0.10, 0.12	
**Laser process parameters**	**Laser power (W)**	**Welding speed (m/min)**
Heat conduction welding (hcw)	5000, 3000	40 60
Stable deep penetration welding (spdw)	6000, 5000 4000	40, 20, 20, 15
Unstable deep penetration welding (updw)	6000, 5000, 4000 3000	10 6

## Data Availability

Not applicable.
